# Comparison of Using One Trabecular Microbypass Stent versus Two during Cataract Surgery at Two Sites: One-Year Follow-Up

**DOI:** 10.1155/2020/1920352

**Published:** 2020-04-07

**Authors:** Shuo Yang, Mantapond Ittarat, Elaine Tran, Patricia Ferrell, Gloria Wang, Ann C. Fisher, Zhongqiu Li, Robert T. Chang

**Affiliations:** ^1^Department of Ophthalmology, Beijing Chao Yang Hospital, Capital Medical University, Beijing, China; ^2^Byers Eye Institute, Stanford University School of Medicine, Palo Alto, CA, USA; ^3^Veterans Affairs Palo Alto Health Care System, Palo Alto, CA, USA

## Abstract

**Purpose:**

To compare IOP and ocular hypotensive medication reduction of using one trabecular microbypass stent versus two in patients with open-angle glaucoma. *Setting*. Palo Alto Veterans Affairs (VA) Hospital and the Byers Eye Institute at Stanford University, Palo Alto, California, USA.

**Design:**

Retrospective case series.

**Methods:**

A chart review included patients who underwent trabecular microbypass implantation with cataract surgery in 2015-2017, with at least one-year follow-up. Subjects were divided into two groups by location (always one stent at Stanford versus two stents at the VA). Primary outcome measures included IOP and medication reduction at baseline and 12-month follow-up.

**Results:**

132 subjects (166 eyes) were included. The preoperative IOP was 16.3 ± 3.4 mmHg on 2.6 ± 1.1 medications in the one-stent group (*N* = 85) and 17.5 ± 3.1 mmHg on 2.7 ± 0.6 medications in the two-stent group (*N* = 81). There was no significant difference between the two groups (*p* = 0.06). At the 12-month visit, there was a 13.37% ± 2.93 reduction in IOP in the 1-stent group (*p* ≤ 0.001) and 13.49% ± 2.69 in the 2-stent group (*p* ≤ 0.001); both were not significantly different from each other (*p* = 0.074). At 12 months, there was also a 14.5% reduction in medication use for the 1-stent group and 15.3% reduction in the 2-stent group, both statistically significant from baseline, (*p* = 0.022 and *p* = 0.037, respectively).

**Conclusions:**

Implantation with either one or two stents during cataract surgery in patients with glaucoma demonstrated similar IOP and med reduction in both groups between the two sites.

## 1. Introduction

Glaucoma is one of the leading causes of irreversible blindness worldwide [1]. Because elevated intraocular pressure (IOP) is a risk factor for progressive optic nerve damage, reducing the IOP by surgical means other than filtration surgery has grown popular in the past decade, particularly as the safety profiles for these methods have improved [2]. This new category of glaucoma procedures, often referred to as minimally invasive glaucoma surgery (MIGS), has provided additional options for ophthalmologists to both restore and/or safely bypass the natural drainage system [3–8]. The first-generation trabecular microbypass stent (Glaukos Corporation, San Clemente, CA, USA), a US FDA-approved implant in 2012, was designed to bypass the main site of aqueous outflow resistance through the trabecular meshwork and lower IOP in mild to moderate open-angle glaucoma (OAG), with minimal trauma to intraocular and extraocular structures. This was the smallest implantable stent to safely reduce both IOP and medication usage and can be placed concurrently with cataract surgery. Samuelson et al. [3] found that 72% of eyes with iStent implantation achieved an unmedicated IOP of less than or equal to 21 mmHg at one year, vs. 50% of eyes in their control group. Neuhann [8] reported a 34% reduction in mean IOP and a 93% reduction in the mean number of medications for patients without prior glaucoma surgery, after three years with the implant. More recently, other studies, like those of Belovay et al. [9], studied the effects of multiple stent placement, reporting a 25% IOP reduction at one year postoperatively for those with two implants and a 26% reduction for those with three. Overall, they reported a 74% decrease in the mean number of medications, with the 3-stent group on significantly fewer medications than the 2-stent group at one year. However, no studies have directly compared results for single versus multiple stent placement directly.

At the Byers Eye Institute, patients with moderate to severe glaucoma received a single stent implant, while those at the Palo Alto Veterans Affairs Hospital received two stents for all patients, which was standard practice since patients did not need to pay extra for the second stent. To study the effects of using one versus two stents at this level of glaucoma severity, we evaluated postoperative IOP and medication reduction between the two institutions.

## 2. Methods

The Byers Eye Institute and the Palo Alto Veterans Affairs records from 2015 to 2017 were retrospectively analyzed. Data for a total of 166 eyes of 132 patients with open-angle glaucoma (POAG) with at least one-year follow-up at both sites were collected. Cases were identified by CPT code 191T. Stanford REDCap system was used to store anonymized data including demographic information, examination findings such as medication usage, cup-to-disc ratio, IOP, pachymetry, visual field mean deviation, and pattern standard deviation. Medication use was extracted from the patient charts, with combination agents counted as separate medications. Surgeries for those in the one-stent group were performed at the Byers Eye Institute (RC, AF), while those of the two-stent group were performed at the Palo Alto Veterans Affairs Hospital (GW, PW). All patients had open-angle glaucoma, including even moderate to severe glaucoma, with visually significant cataracts, and had undergone surgeries between 2015 and 2017 at one of these two sites. Glaucoma severity was defined by standard 24-2 automated perimetry (SAP) using Hodapp-Parrish-Anderson staging as mild glaucoma with mean deviation (MD) within 0 to -6 dB, moderate glaucoma with MD within -6 to -12 dB, and severe glaucoma with MD greater than -12 dB. Patients were excluded if they had any prior glaucoma surgery, angle closure, or traumatic, neovascular, or uveitic glaucoma. Preoperative IOP and medication usage were taken from the latest preoperative visit.

The iStent itself is a titanium-made, heparin-coated, single-piece design. The stent measures 1.0 mm in length and 0.33 mm in height, with a snorkel length of 0.25 mm and a diameter of 120 *μ*m. The snorkel on the short side of the L-shaped structure resides in the anterior chamber and opens to a half-pipe body, which resides in Schlemm's canal. There are two model numbers (R and L) available, correlating to a right-flow stent and left-flow stent, respectively. After standard phacoemulsification and intraocular lens (IOL) implantation, all four surgeons used the same technique with an open-access gonio lens to view the angle under a viscoelastic solution. At the Byers Eye Institute, one stent was gently placed inferonasally through the trabecular meshwork and into Schlemm's canal based on the targeted placement of limbal vasculature (to increase likelihood of connecting with a collector channel) and/or the most pigmented region of the TM. In the Palo Alto Veterans Affairs Hospital, two stents were placed successfully either inferiornasally or superiornasally (depending on left- or right-handedness of the surgeon. All cases included in the study had successful placement without complication by experienced surgeons. Patients returned to the clinic for examination at postoperative day one, week one, and routine follow-up to one year. Postoperative assessment included best-corrected visual acuity, IOP, medication usage, and structural and functional testing. Primary outcome measures were postoperative IOP and number of glaucoma medications at the one-month, three-month, and 12-month follow-up. Statistical tests were performed using GraphPad Prism 5.01 (GraphPad Prism Software, Inc.). Within each group, paired-sample *t*-tests were used to compare changes in IOP and the number of medications at one, three, and 12 months for both one- and two-stent groups. One-way ANOVA and nonparametric *t*-tests were used to compare between different groups at different time points.

This study was conducted in accordance with the tenets of the Declaration of Helsinki, and the protocol was approved by the Stanford University and Palo Alto Veterans Affairs Institutional Review Board. Retrospective chart review was conducted at two sites: the Byers Eye Institute at Stanford and the Palo Alto Veterans Affairs Hospital.

## 3. Results

### 3.1. Subject Demographics and Preoperative Parameters

A total of 166 eyes of 132 patients with cataracts and POAG were implanted with either one or two microbypass stents during cataract surgery. In this case series, 96 patients were male and 36 were female, and the average age was 76 ± 9 years old. The cup-to-disc ratio (C : D) was 0.8 ± 0.1. The preoperative pachymetry was 528 ± 42 *μ*m. Subject demographics and parameters are summarized in Table 1.

### 3.2. Preoperative IOP and Medication Use

**Figure 1:**
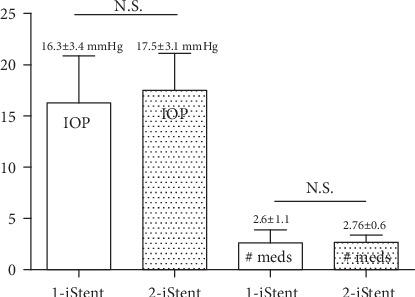
Preoperative IOP and medication number. The solid bars represent the one-stent group, and the dotted bars represent the two-stent group.

Preoperative IOP with medication and total medication use distribution of the two groups are provided in Figure 1. In the one-stent group, the preoperative IOP was 16.3 ± 3.4 mmHg, and in the two-stent group, the preoperative IOP was 17.5 ± 3.1 mmHg. There was no significant difference in preoperative IOP between the two groups (*p* = 0.06). The number of preoperative medications in the one-stent group was 2.6 ± 1.1 and 2.7 ± 0.6 in the two-stent group. There was also no significant difference in preoperative medication use between the two groups (*p* = 0.36).

**Figure 2:**
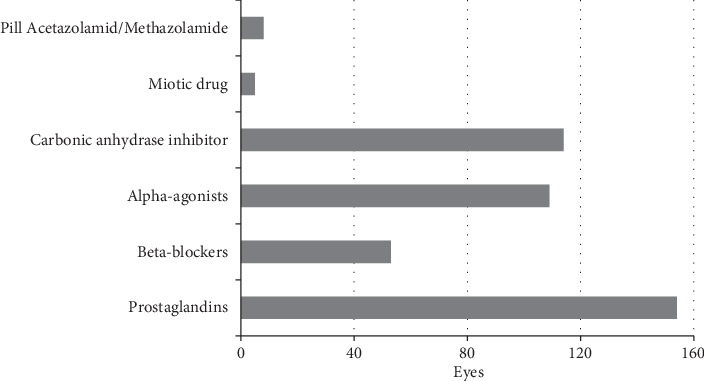
Preoperative medications. Up to 6 kinds of glaucoma medications were counted.

Preoperative medication use in the two groups is shown in Figure 2. In total, 92.8% used prostaglandins (154), 31.9% beta-blockers (53), 65.7% alpha-agonists (109), 68.7% carbonic anhydrase inhibitor (114), 3.0% pilocarpine (5), and 4.8% oral carbonic anhydrase inhibitor pill, either acetazolamide or methazolamide (8).

### 3.3. Postoperative Assessment

**Figure 3:**
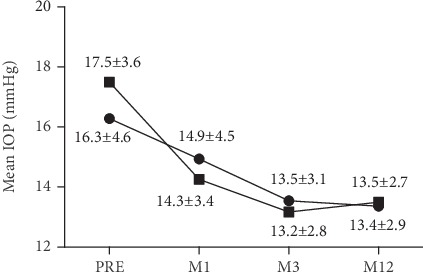
Postoperative IOP. The one-stent group values are shown in solid circles, while those of the two-stent group are in solid squares.

**Figure 4:**
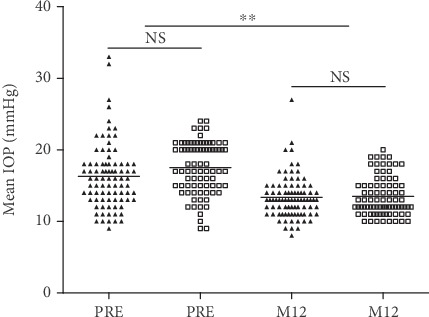
Baseline IOP and postoperative IOP at month 12. The one-stent group values are shown in solid triangles, while those of the two-stent group are in hollow squares, ^∗∗^<0.001, compared between the two groups and between pre- and postoperation.

As shown in Figures 3 and 4, the mean IOP in the one-stent group decreased. At month 1 follow-up, the mean IOP was reduced from 16.3 ± 4.6 mmHg to 14.9 ± 4.5 mmHg. At month 3, the mean IOP was 13.5 ± 3.1 mmHg, and at month 12, it was 13.4 ± 2.9 mmHg. The postoperative mean IOP were significantly lower than the preoperative IOP (*p* = 0.046, *p* ≤ 0.001, and *p* ≤ 0.001). There was a 17.8% reduction of the mean IOP at month 12 compared with the preoperative mean IOP. 37.2% of eyes had a 20% IOP reduction at 12 months following surgery. In the two-stent group, the mean IOP at month 1, month 3, and month 12 was 14.3 ± 3.4 mmHg, 13.2 ± 2.8 mmHg, and 13.5 ± 2.7 mmHg, respectively. The postoperative mean IOP in the two stent-group were significantly lower than the preoperative IOP values (*p* ≤ 0.001). At month 12, there was a 22.8% mean IOP reduction compared with the preoperative IOP, and 64.1% of eyes had an IOP reduction ≥ 20%. And at month 12, 71 eyes (83.5%) in the one-stent group achieved an IOP ≤ 15 mmHg, and 63 eyes (77.8%) in the two-stent group achieved an IOP ≤ 15 mmHg. There were no significant differences in the mean IOP between the one-stent group and the two-stent group at any postoperative time point (*p* > 0.05, respectively).

**Figure 5:**
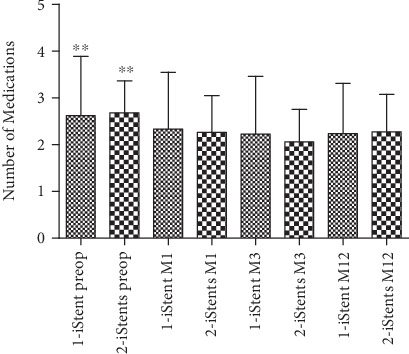
Comparison of medication number. The one-stent group is represented by dotted bars, and the two-stent group by checkered bars, ^∗∗^<0.001, compared among all time points.

Medications used by each group are provided in Figure 5. In both the one-stent group and the two-stent group, the number of postoperative medication used was less than that of the preoperative medication used (*p* ≤ 0.001). There was a 15% reduction of the number of medication use in both groups after surgery. There were no significant differences of the number of postoperative medication usage between the one-stent and two-stent groups at any time point (*p* = 0.64).

## 4. Discussion

Prior studies have evaluated single [3–8] and multiple [9–11] iStent implantation during cataract surgery in open-angle glaucoma patients. Prospective randomized studies of single stent placement, including those of Samuelson et al. [3] (240 eyes), Fea [4] (33 eyes), and Craven et al. [5] (240 eyes), found better pressure reduction for patients who underwent cataract surgery with stent implantation, rather than cataract surgery alone, on fewer medications. Samuelson et al. [3] found a significant 1.5 mmHg IOP reduction following cataract surgery with stent implantation as well as a reduction of 1.4 in the mean number of medication use over a 1-year period. Fea [4] found an average IOP reduction of 3.1 mmHg for those who underwent stent implantation with cataract surgery, with a mean reduction of 1.6 in medication at 15 months. Craven et al. [5] reported a 1.6 mmHg IOP reduction and 2.0 reduction in mean medication over a two-year period. Similarly, the uncontrolled studies of Arriola-Villalobos et al. [6] (19 eyes) and Spiegel et al. [7] (47 eyes) also found combined cataract surgery and iStent placement to be an effective means of lowering IOP pressure with minimal medication for patients with coexisting open-angle glaucoma and cataract. Arriola-Villalobos et al. [6] studied patients over 3 years, reporting a mean 3.16 mmHg in IOP reduction in iStent-treated patients and a mean reduction of 0.48 in medication. Spiegel et al. [7] (47 eyes) reported 4.4 mmHg IOP reduction and a mean decrease in mean medication by 1 over a 1-year period. Studies of multiple iStent placement report mean IOP and medication use reductions of similar magnitude to these single iStent implantation studies. Belovay et al.'s [9] comparative study (53 eyes) found both 2 and 3 iStent trabecular microbypass stent placements to reduce mean IOP by 3.7 mmHg as well as mean medication use by 2.0. These separate studies report comparable outcomes for either single or multiple stent placement, but it remains to be seen how these two therapies compare side by side in the same analysis.

In the present study, we compared postoperative IOP and medication changes between a group at one site receiving one stent and another group at a different site receiving two stents in patients with moderate to severe glaucoma. At the postoperative 12-month point, there was a mean IOP reduction of 17.8% in the one-stent group, and 22.8% in the two-stent group. Both groups exhibited nearly 15% medication reduction compared with preoperative medication use. While our study describes a modest IOP and medication reduction following iStent surgery, the extent of IOP and medication changes was not as great as those reported by Lindstrom et al. and Berdahl et al., who report a 30%-40% IOP reduction [10, 11]. Compared with their studies, our groups had a lower baseline IOP; it is reasonable that their studies had a higher percent of IOP reduction due to a higher baseline. We think that MIGS has a definite level of postoperative IOP. In addition, we found no significant differences either in postoperative IOP or in medication use between the two groups.

There were a number of limitations to this study, including that it was retrospective and involved four different surgeons at two different sites; therefore, the technique may differ. Though the iStent is approved for treatment of mild to moderate glaucoma, our study involved also patients with severe glaucoma as only these patients were considered for treatment with two stents. Also, we did some iStent on ocular hypertensive patients if not tolerating drops. While the level of severity was kept similar between sites, patient populations may still differ as the Veterans Affairs Hospital treats a select, mostly male population. We did not show a more detailed range of severity in our cohort. The wide MD had to do with the severity of cataract. We could not relate visual acuity or cataract stage to MD well.

Despite these challenges, this study is the first of its kind to directly compare mean IOP and medication use in patients treated with one versus two stents between two different sites. As to the optimal numbers of stents implanted, there are currently few studies that have elucidated the difference in efficacy of one versus multistents in lowering intraocular pressure and reducing the need for glaucoma medications. Though the postoperative mean IOP values were significantly lower than preoperative IOP values in each group, we found no significant difference in reduction in mean IOP between groups. Different from other similar studies, such as the study by Katz et al. [12], our results suggest that an additional stent implantation may not culminate in additive or synergistic effects on postoperative IOP. We surmise that the additive effect of aqueous fluid outflow may be not remarkably increased with an additional stent implantation. Our study encompassed data only through 12 months and is not as long as the study by Katz et al. Also, different sites may have potential bias, for all patients treated with two stents are from the Veterans Affairs Hospital and mostly male. Our study also suggests that treatment with either one or two stent placement, even in the setting of variable settings, systems, and surgical skill, can be effective in treating moderate to severe glaucoma. Further longer-term and accurate studies would be helpful in establishing extended surgical indications for implantation of trabecular microbypass stents in the future.

Combined trabecular microbypass stents and cataract procedures have been shown to safely and effectively treat those with open-angle glaucoma and clinically significant cataracts. In this large cohort retrospective review comparing iStent surgical results between those who received one stent and those who received two, we honestly report that little difference was found in postoperative IOP reduction or medication use in patients with moderate to severe glaucoma. In an era of rising health care costs, our study suggests that further exploration into the value of multiple stent implantation may inform more cost-effective glaucoma treatment practices. The postoperative medication use was a subjective and nonprotocol decision. Whether one or two stents were implanted, the glaucoma medication cannot be completely discontinued for glaucoma doctors always want to see stable and low postoperative pressure and do not want to stop patients' medications rashly.

## Figures and Tables

**Table 1:** Subject demographic and preoperative characteristic.

Total	166 eyes of 132 subjects
Hispanic/Latino	21.2%
Non-Hispanic/Latino	75%
Not reported	3.8%
Asian/Pacific Islander	19.7%
Black	12.9%
White	65.9%
Gender (male/female)	96/36
Age (years)	75.77 ± 8.83
Cup-to-disc (C : D) ratio	0.81 ± 0.11
Preoperative pachymetry	527.89 ± 41.69
Visual field mean deviation (dB)	5.29 ± 8.91
Visual field pattern standard deviation	8.17 ± 3.63

Ethnicity and race reported as percentages of the total sample (*N* = 132).

## Data Availability

The data used to support the findings of this study are restricted by Veterans Affairs Palo Alto Health Care System confidential policy in order to protect soldiers' privacy. Data are available from Robert T. Chang (Byers Eye Institute, Stanford University School of Medicine, Palo Alto, CA, USA) and Gloria Wang(Veterans Affairs Palo Alto Health Care System, Palo Alto, CA, USA).

## Supplementary Materials

Supplementary Materials The supplementary material file is the image of istent we describe in the manuscript.
