# Supplementary figures and images for: Comparison of Using One Trabecular Microbypass Stent versus Two during Cataract Surgery at Two Sites: One-Year Follow-Up
Source: Biomed Res Int. 2020 Apr 7;2020:1920352. doi: 10.1155/2020/1920352 (PMC7166285; doi:10.1155/2020/1920352)

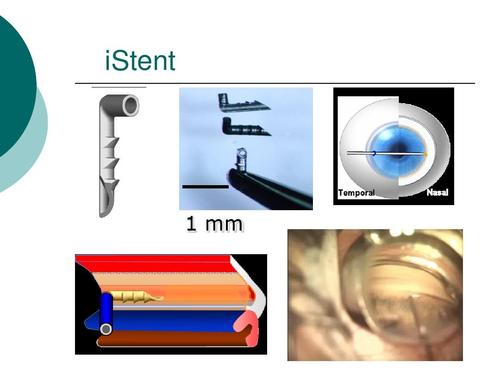

Supplement: Supplementary Materials — The supplementary material file is the image of istent we describe in the manuscript. [file 1920352.f1.jpg]
